# Effects of Nitrogen Level during Seed Production on Wheat Seed Vigor and Seedling Establishment at the Transcriptome Level

**DOI:** 10.3390/ijms19113417

**Published:** 2018-10-31

**Authors:** Daxing Wen, Haicheng Xu, Liuyong Xie, Mingrong He, Hongcun Hou, Chenglai Wu, Yan Li, Chunqing Zhang

**Affiliations:** State Key Laboratory of Crop Biology, Agronomy College, Shandong Agricultural University, Tai’an 271018, China; daxing100@126.com (D.W.); xuhaich@126.com (H.X.); xieliuyong10@163.com (L.X.); mrhe@sdau.edu.cn (M.H.); houhongcun11@163.com (H.H.); clwu@sdau.edu.cn (C.W.); yanli@sdau.edu.cn (Y.L.)

**Keywords:** nitrogen, seed production, seed vigor, seedling establishment, wheat

## Abstract

Nitrogen fertilizer is a critical determinant of grain yield and seed quality in wheat. However, the mechanism of nitrogen level during seed production affecting wheat seed vigor and seedling establishment at the transcriptome level remains unknown. Here, we report that wheat seeds produced under different nitrogen levels (N0, N168, N240, and N300) showed significant differences in seed vigor and seedling establishment. In grain yield and seed vigor, N0 and N240 treatments showed the minimum and maximum, respectively. Subsequently, we used RNA-seq to analyze the transcriptomes of seeds and seedlings under N0 and N240 at the early stage of seedling establishment. Gene Ontology (GO) term enrichment analysis revealed that dioxygenase-activity-related genes were dramatically upregulated in faster growing seedlings. Among these genes, the top three involved linoleate 9S-lipoxygenase (*Traes_2DL_D4BCDAA76*, *Traes_2DL_CE85DC5C0*, and *Traes_2DL_B5B62EE11*). Kyoto Encyclopedia of Genes and Genomes (KEGG) enrichment analysis revealed that pathways involved in nutrient mobilization and the antioxidant system showed enhanced expression under N240. Moreover, seeds with faster growing seedlings had a higher gene expression level of α-amylase, which was consistent with α-amylase activity. Taken together, we propose a model for seedling establishment and seed vigor in response to nitrogen level during seed production.

## 1. Introduction

Seed vigor, an important index of seed quality, determines the potential for the rapid and uniform emergence of plants [1]. In agricultural production, high seed vigor is connected with the potential for increasing growth and productivity [2]. Seeds acquire vigor during the maturation drying stage in seed development, which is not only involved in the water loss process but also a remarkable proportion of the gene expression and metabolic signatures similar to those that characterize seed germination [3]. A standard germination test that includes two phases (germination and seedling establishment) can be used for evaluating seed vigor. Studies on seedling establishment and seed vigor involve many aspects, such as physiological traits, genes, and “-omics” levels [4,5,6,7]. At present, searching for physiological and molecular indicators of seed vigor is a pivotal goal for basic and applied research [8].

Seedling establishment is critical in the life cycle of plants after seed germination [6] and requires reserve remobilization to supply nutrients [9]. Wheat (*Triticum aestivum* L.) seeds contain many kinds of storage reserves, such as starches, lipids, and proteins. These large, insoluble storage reserves need to be converted into smaller metabolites, such as sucrose, which can be transported to the developing organs [10]. However, which kinds of storage reserves play crucial roles in seedling establishment in response to nitrogen level during seed production are still unknown.

Genotype, environmental conditions during seed development, and storage conditions impact seedling establishment and seed vigor. Genome-wide association studies (GWAS) and quantitative trait loci (QTL) was used to study seed-vigor-related traits [2,11,12,13,14]. Dehydration and rehydration during seed development and germination are associated with high levels of oxidative stress, resulting in DNA damage. Loss of seed vigor during storage is mainly affected by seed moisture content and storage temperature. Free radicals and lipid peroxidation are widely considered as the primary cause of decreased seed vigor [15]. Low-vigor seeds can be improved by many approaches, such as seed priming, electric field treatment, and magnetic field treatment [16,17,18]. Extensive studies show that nitrogen affects various aspects of the life cycle of wheat, such as growth, grain yield, seed composition, and seedling performance [19,20,21,22,23]. However, the effects of nitrogen level during seed production on seed vigor and seedling establishment at the transcriptome level remain poorly understood.

In this study, wheat seeds produced under different nitrogen levels showed significant differences in seed vigor and seedling establishment. Moreover, we used RNA-seq to analyze seedling establishment differences at the transcriptome level. Pathways involved in nutrient mobilization and the antioxidant system were dramatically downregulated in seedling establishment under a low nitrogen level. Taken together, the results provide new insights into seedling establishment and seed vigor in response to parental nitrogen level.

## 2. Results

### 2.1. Nitrogen Level during Seed Production Significantly Affects Seed Vigor

**Figure 1:**
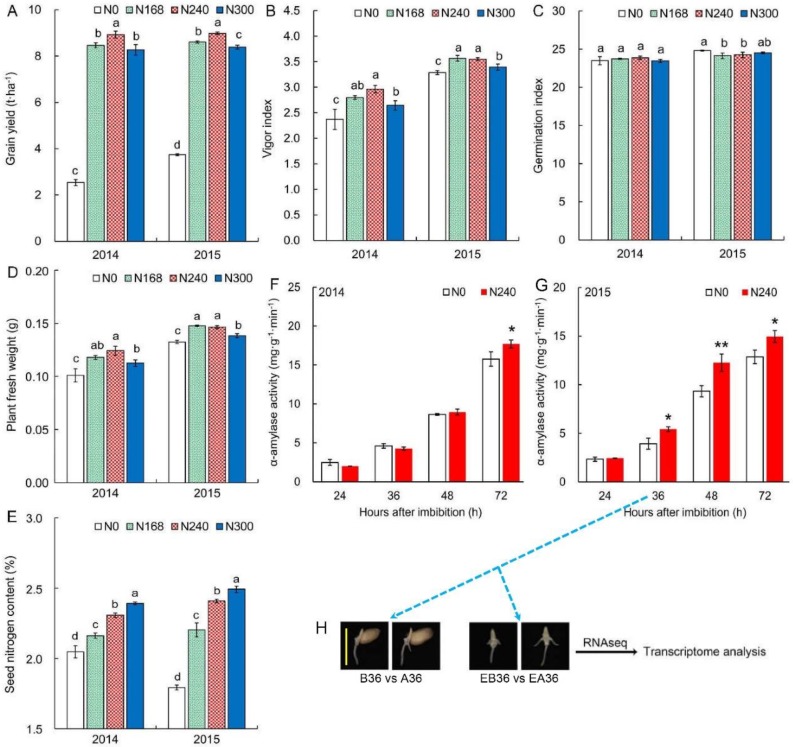
Effects of nitrogen fertilizer on wheat grain yield and seed vigor over two years for four nitrogen fertilizer levels: N0 (0 kg/ha), N168 (168 kg/ha), N240 (240 kg/ha, the usual nitrogen fertilizer level for winter wheat production in the North China Plain), and N300 (300 kg/ha): (**A**) grain yield came from field plot experiment; (**B**) vigor index; (**C**) germination index; (**D**) plant fresh weight came from standard germination test; (**E**) seed nitrogen content; (**F**,**G**) α-amylase activity under N0 and N240 from 24 to 72 HAI; and (**H**) two kinds of sampling methods in transcriptome analysis. B36 vs. A36: Samples (seeds and seedlings) under N240 compared to those under N0 at 36 HAI. EB36 vs. EA36: Samples (only seedlings) under N240 compared to those under N0 at 36 HAI. Scale bar, 1 cm. Error bars represent the standard deviation for three field plot replicates and each field plot replicate includes at least three technical replicates. Different letters indicate significant differences among means under different treatments (*p* value < 0.05 by one-way ANOVA analysis). Asterisks denote a significant difference according to an unpaired Student’s *t*-test (*: *p* < 0.05; **: *p* < 0.01).

To study the effects of nitrogen fertilizer during seed production on seed vigor and seedling establishment, we set four nitrogen levels: N0 (0 kg/ha), N168 (168 kg/ha), N240 (240 kg/ha, the usual nitrogen fertilizer level for winter wheat production in the North China Plain), and N300 (300 kg/ha). N240 and N0 treatments showed the biggest and smallest grain yields over two years, respectively (Figure 1A). We evaluated seed vigor by the vigor index, which equals the germination index multiplied by plant fresh weight. There were no obvious differences between N168 and N240 in the vigor index, but the two treatments had remarkably higher vigor indexes than N0 and N300 over two years (Figure 1B). However, what was the reason seeds showed different vigor under different nitrogen levels during seed production? The germination index showed similar trends under four nitrogen levels over two years, and there were no significant differences among the four nitrogen levels in 2014 (Figure 1C). Seeds under N168 and N240 had slightly lower germination indexes than seeds of N0 and N300 in 2015. There were no obvious differences between N168 and N240 in plant fresh weight, but the two treatments had slightly higher plant fresh weights than N0 and N300 over two years (Figure 1D). Therefore, the vigor index was mainly determined by plant fresh weight when the germination index was similar among treatments. These results indicated that nitrogen level affected not only grain yield but also seed vigor and seedling establishment. Seed nitrogen content enhanced dramatically following the increase of nitrogen level (Figure 1E). Seeds under N300 displayed the highest seed nitrogen content over two years, but seed vigor under N300 was lower than that under N240. Therefore, seed nitrogen content could not reflect seed vigor and seedling establishment accurately, which might be due to seed composition having a complex influence on seed vigor and seedling establishment. Besides nitrogen metabolism at seedling establishment, there might be other metabolism pathways in response to nitrogen level.

Considering high seed vigor and high grain yield, we used seeds under N240 to analyze the reason for seedling establishment differences compared with seeds under N0 in the subsequent experiments. As the main storage material in wheat, starch can supply energy and a material base for seedling growth. Starch decomposition needs a series of hydrolases and α-amylase that play an important role in seedling establishment. The activity of α-amylase was relative lower at germination 24 h after imbibition (HAI). Most seeds’ radicles broke through the seed coat at this moment and then α-amylase activity gradually increased from germination 24–72 HAI (Figure 1F,G). For α-amylase activity, N0 seeds were remarkably lower than N240 seeds at germination 72 HAI in 2014 and from germination 36–72 HAI in 2015, respectively. Therefore, α-amylase activity at 72 HAI could reflect the vigor index.

### 2.2. Comparison of Gene Expression in Seedling Establishment at Early Stage

To explore genes and gene networks that control wheat seedling establishment in response to parental nitrogen level, we selected samples at 36 HAI in 2015 (α-amylase activity began to show differences at this moment) for transcriptome analysis of seedling establishment (Figure 1H). We used two kinds of sampling methods (Figure 1G). The first one was samples (seeds and seedlings) under N240 compared to those under N0 at 36 HAI (B36 vs. A36), which was used for exploring the interaction between seed and seedling during seedling establishment (mainly involved in nutrient mobilization from seed to seedling). The second one was samples (only seedlings) under N240 compared to those under N0 at 36 HAI (EB36 vs. EA36), which was used for investigating the differences among seedlings at the transcriptome level. RNA-seq generated 41–58 million reads for each sample, and each treatment included three biological replicates (Table S1). After removing low quality regions and adapter sequences, there remained about 39–56 million clean reads. Subsequently, about 30–38 million clean reads were mapped to the wheat genome. In each library, 65.37–66.92% of clean reads were mapped to single locations in the reference sequence (uniquely mapped reads), and 9.75–10.12% clean reads were mapped to multiple locations (multiple mapped reads). The R package edgeR was used to identify differentially expressed genes (DEGs) by using a false discovery rate of <0.05 as the significance cutoff. We found that 235 genes were significantly upregulated and 180 genes were significantly downregulated in B36 vs. A36, and 306 genes were significantly upregulated and 210 genes were significantly downregulated in EB36 vs. EA36.

### 2.3. Dioxygenase-Activity-Related Genes Are Involved in Seedling Establishment Regulation

**Figure 2:**
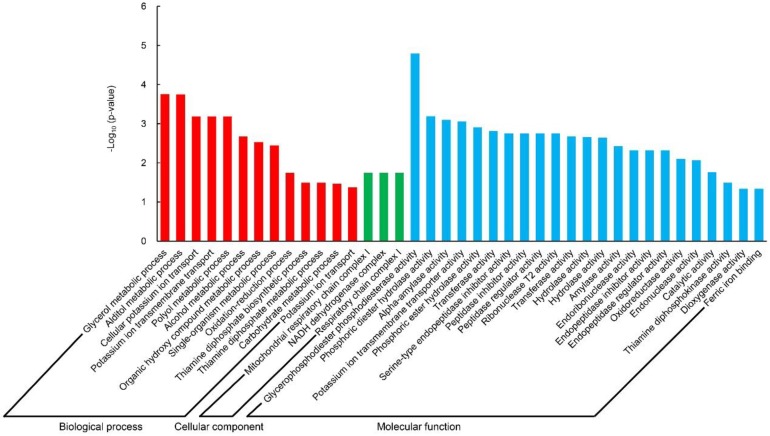
Significantly enriched Gene Ontology (GO) terms (*p* < 0.05) analysis in B36 vs. A36. B36 vs. A36: Samples (seeds and seedlings) under N240 compared to those under N0 at 36 HAI.

To understand the function of these DEGs, Gene Ontology (GO) term enrichment analysis (*p* < 0.05) was performed. In B36 vs. A36, the most remarkably enriched GO term was the glycerol metabolic process (GO: 0006071, *p* = 1.76 × 10^−4^) in the biological process group, mitochondrial respiratory chain complex I (GO: 0005747, *p* = 1.79 × 10^−2^) in the cellular component, and glycerophosphodiester phosphodiesterase activity (GO: 0008889, *p* = 1.62 × 10^−5^) in the molecular function group, respectively (Figure 2). Glycerol metabolic process (GO: 0006071) and glycerophosphodiester phosphodiesterase activity (GO: 0008889) showed the same DEGs, including six upregulated genes and one downregulated gene (Table 1). These results indicated that glycerol metabolism under N240 was significantly higher at 36 HAI than that under N0. Moreover, we also found that the GO term alpha-amylase activity (GO: 0004556, *p* = 7.93 × 10^−4^) was enriched in the molecular function group, suggesting that N240 seeds might have higher starch decomposition ability at 36 HAI than N0 seeds.

**Figure 3:**
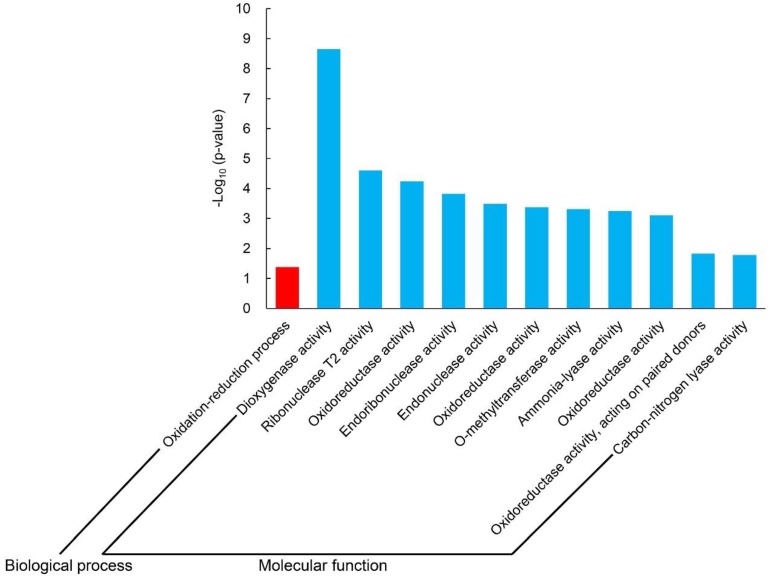
Significantly enriched Gene Ontology (GO) terms (*p* < 0.05) analysis in EB36 vs. EA36. EB36 vs. EA36: Samples (only seedlings) under N240 compared to those under N0 at 36 HAI.

In EB36 vs. EA36, the most remarkably enriched GO term was the oxidation-reduction process (GO: 0055114, *p* = 1.16 × 10^−4^) in the biological process group and dioxygenase activity (GO: 0051213, *p* = 5.21 × 10^−13^) in the molecular function group (Figure 3). The oxidation-reduction process and dioxygenase activity are related to material conversions that are vital for seedling establishment. Dioxygenase activity (GO: 0051213, *p* = 5.21 × 10^−13^) was the most significantly enriched GO term in EB36 vs. EA36, indicating that dioxygenase activity played a pivotal role in seedling establishment. DEGs involved in dioxygenase activity included 23 upregulated genes and one downregulated gene (Table 2). The top three DEGs involved linoleate 9S-lipoxygenase (*Traes_2DL_D4BCDAA76*, *Traes_2DL_CE85DC5C0*, and *Traes_2DL_B5B62EE11*). *Traes_2DL_D4BCDAA76* showed an 11-fold (log2Foldchange = 3.49) higher expression in B36 vs. A36. Moreover, phytohormone-biosynthesis-related genes were also enriched in dioxygenase activity. DEGs of 1-aminocyclopropane-1-carboxylate oxidase (*Traes_3DL_441FB3597* and *Traes_2DS_6789FA5E7*) are involved in the ethylene biosynthesis, and gibberellin 20 oxidase 1 (*Traes_4DS_A89A8FAD91* and *Traes_4BS_A772DDBD7*) participates in the biosynthesis of gibberellin. Overall, dioxygenase activity (GO: 0051213) was significantly upregulated in EB36 vs. EA36.

### 2.4. Pathways Involved in Nutrient Mobilization and Glutathione Metabolism Show Enhanced Expression in Faster Growing Seedlings

**Figure 4:**
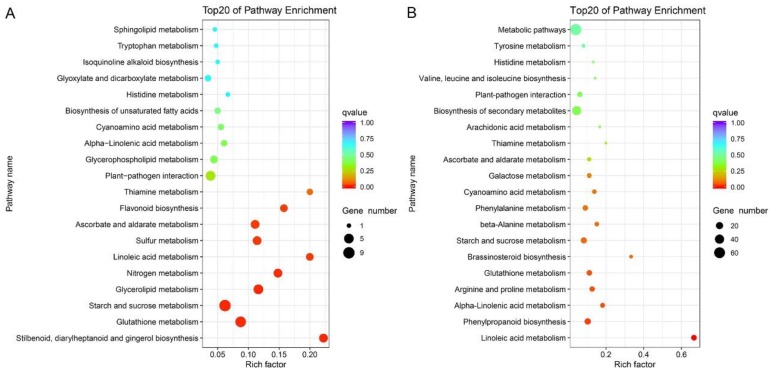
Top 20 Kyoto Encyclopedia of Genes and Genomes (KEGG) pathways in: B36 vs. A36 (**A**); and EB36 vs. EA36 (**B**). B36 vs. A36: Samples (seeds and seedlings) under N240 compared to those under N0 at 36 HAI. EB36 vs. EA36: Samples (only seedlings) under N240 compared to those under N0 at 36 HAI.

To investigate the differences of metabolism pathways, we also analyzed the Kyoto Encyclopedia of Genes and Genomes (KEGG) enrichment. Stilbenoid, diarylheptanoid, and gingerol biosynthesis (ko00945, *p* = 1.13 × 10^−2^) was the most significantly enriched pathway in B36 vs. A36 (Figure 4A). In this pathway, the biggest fold change of expression level was *Traes_4AL_89EAC5E10* (log2Foldchange = 1.5286, annotation: tricin synthase). Moreover, a near *p*-value of enriched pathway in B36 vs. A36 was glutathione metabolism (ko00480, *p* = 1.13 × 10^−2^), which was also enriched in EB36 vs. EA36 (Figure 4B). Glutathione metabolism is related to scavenging of reactive oxygen species (ROS). In glutathione metabolism, most DEGs in B36 vs. A36 and EB36 vs. EA36 were upregulated except *TRAES3BF053500110CFD_g* and *TRAES3BF156400110CFD_g* in B36 vs. A36 and *Traes_1AS_CBD6D1EA5* in EB36 vs. EA36 (Table S2). FPKM (expected number of Fragments per Kilobase of transcript sequence per Million base pairs sequenced), which is consistent with gene expression level, needed to be considered when an enzyme had more than one DEG. For instance, glutathione S-transferase [EC: 2.5.1.18] in EB36 vs. EA36 had two DEGs, *Traes_1AS_CBD6D1EA5* and *Traes_4DL_469179461*, and the former had lower FPKM than the latter (Table S3). Thus, *Traes_4DL_469179461* played an important role in glutathione S-transferase [EC: 2.5.1.18] in EB36 vs. EA36, and glutathione S-transferase showed upregulation. Moreover, all of the DEGs of l-ascorbate peroxidase [EC: 1.11.1.11] showed upregulation and had higher FPKM than that in glutathione-related enzymes. Overall, N240 seeds had higher glutathione metabolism than N0 seeds at 36 HAI.

Several pathways involved in nutrient mobilization were enriched in B36 vs. A36, such as starch and sucrose metabolism (ko00500, *p* = 1.16 × 10^−2^), nitrogen metabolism (ko00910, *p* = 1.36 × 10^−2^), and linoleic acid metabolism (ko00591, *p* = 2.18 × 10^−2^) (Figure 4A). The leading storage reserve in wheat is starch, which supplies nutrients for seedling establishment. Subsequently, we analyzed starch and sucrose metabolism. Overall, starch and sucrose metabolism under N240 was higher at 36 HAI than that under N0 (Table S3). DEGs in B36 vs. A36 were mainly involved in starch catabolism. Sucrose and cell wall metabolism played important roles in EB36 vs. EA36. Alpha-amylase [EC: 3.2.1.1] had five DEGs (*Traes_6BL_8115FDC31*, *Traes_6DL_E31AB6EED*, *Traes_6DL_5BE701A64*, *Traes_7AL_3C8D25CCD*, and *Traes_7BL_0687BD4F8*), and the former three DEGs had higher FPKMs than the latter two DEGs. Thus, the former three DEGs played a leading role in α-amylase [EC: 3.2.1.1] in B36 vs. A36, and α-amylase showed upregulation. Therefore, the gene expression level of α-amylase was consistent with α-amylase activity. Moreover, DEGs of starch phosphorylase [EC: 2.4.1.1] were also upregulated in B36 vs. A36. Thus, the two pathways of starch decomposition played important roles in seedling establishment in response to parental nitrogen level. In nitrogen metabolism, DEGs of glutamine synthetase (Traes_4DS_47A04A098) and glutamate dehydrogenase (Traes_5DL_FCD84F216) were upregulated in B36 vs. A36 and EB36 vs. EA36, respectively. In linoleic acid metabolism, 3 DEGs were enriched in B36 vs. A36 and 10 DEGs were enriched in EB36 vs. EA36. All of the DEGs were upregulated. These results indicated that pathways involved in nutrient mobilization were enhanced in seedling establishment under N240 at 36 HAI.

### 2.5. Validation of RNA-Seq Data

**Figure 5:**
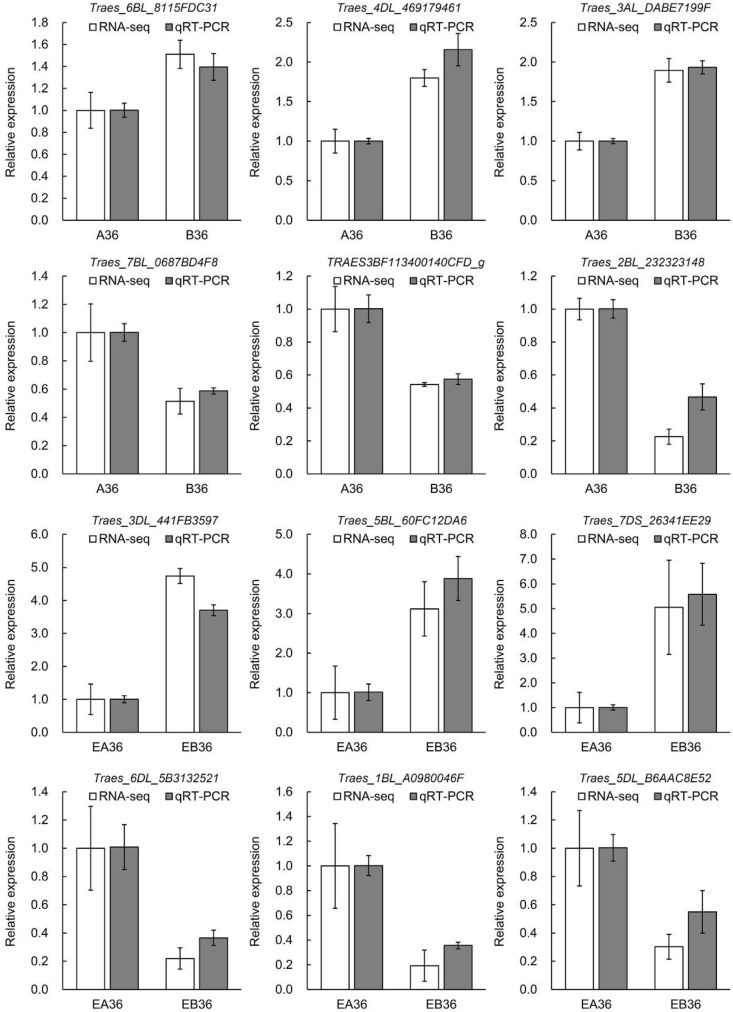
Validation of differentially expressed genes by qRT-PCR. The relative expression level of each gene was expressed as the fold change between N0 and N240 in the RNA-seq data (white bar) and qRT-PCR data (gray bar). The wheat *Actin* gene was used as an internal control to normalize the expression data. Error bars represent the standard deviation for three replicates.

To validate the DEGs identified by high-throughput RNA-seq, we randomly selected 12 DEGs to perform quantitative real-time PCR (qRT-PCR) assays. In these DEGs, three DEGs showed upregulation and three DEGs displayed downregulation in B36 vs. A36 and EB36 vs. EA36, respectively (Figure 5). All 12 DEGs showed similar expression patterns in the qRT-PCR assays as their transcript abundance changes were identified by RNA-seq, which indicated that the RNA-seq data were credible.

## 3. Discussion

Nitrogen fertilizer is critical for grain yield and seed quality in many crops, such as wheat, rice, and maize [19,24,25]. Moreover, a previous study reported changes of metabolism pathways in rice seedling roots in response to nitrogen deficiency [26]. However, effects of nitrogen level during seed production on seed vigor are largely unknown in wheat. In this study, the nitrogen fertilizer level during wheat seed production significantly affected both seed vigor and seedling establishment. Transcriptome analysis revealed that genes and metabolism pathways participated in seedling establishment in response to parental nitrogen level.

α-amylase plays an important role in native starch granule degradation, and its expression is affected by gibberellin (GA) and sugar starvation [27]. Overall, the expression of α-amylase genes was consistent with α-amylase activity at 36 HAI. Although α-amylase activity at 36 HAI in 2014 was different from that in 2015, the overall trend of α-amylase activity was similar over two years. The reason might be the changes of 1000-grain weight over two years [28], thereby resulting in variations in α-amylase activity. α-amylase activity at 72 HAI was consistent with seed vigor over two years, which suggested that α-amylase activity at 72 HAI could be used to rapidly evaluate wheat seed vigor.

Glycerophosphodiester phosphodiesterase (GDPD) hydrolyzes glycerophosphodiesters into alcohols and glycerol-3-phosphate (G-3-P), which play important roles in multiple physiological processes in plants [29,30]. In this study, most DEGs involved in glycerophosphodiester phosphodiesterase showed enhanced expression levels (Table 1), which indicated that multiple physiological processes might have higher metabolism in B36 vs. A36. Dioxygenase is implicated in many kinds of plant hormone metabolism, such as auxin, gibberellin (GA), and abscisic acid (ABA) [31,32,33]. In this study, many upregulated DEGs related to ethylene and GA biosynthesis were enriched in dioxygenase activity in EB36 vs. EA36. Moreover, the top three DEGs in dioxygenase activity in EB36 vs. EA36 encode linoleate 9S-lipoxygenase, which is involved in jasmonic acid (JA) biosynthesis [26]. These results suggest that ethylene, GA, and JA in seedling establishment might participate in the response to parental nitrogen fertilizer level.

The two substrates of tricin synthase (EC: 2.1.1.175) are S-adenosyl-l-methionine and tricetin, whereas its two products are S-adenosyl-l-homocysteine and 3′, 5′-O-dimethyltricetin (tricin). Tricin is an O-methylated flavone, a type of flavonoid. Previous studies have shown that tricin has many functions, such as antioxidant activity and lignification in monocots [34]. In this study, *Traes_4AL_89EAC5E10* (annotation: tricin synthase) enriched in stilbenoid, diarylheptanoid, and gingerol biosynthesis (ko00945) showed enhanced expression in B36 vs. A36. Moreover, glutathione metabolism was a near *p*-value of enriched pathway in B36 vs. A36. These results indicated that antioxidant activity might be higher under N240 than under N0 at 36 HAI.

Ascorbate peroxidases (APXs) involved in glutathione metabolism can reduce H_2_O_2_ escaping from peroxisomes to protect the closely associated oil bodies during seedling growth [35]. In this study, N240 seeds had higher glutathione metabolism than N0 seeds at 36 HAI. l-ascorbate-peroxidase-related [EC:1.11.1.11] DEGs showed about a 10–100-fold higher expression level (FPKM) compared with glutathione-related DEGs (glutathione synthase [EC:6.3.2.3], glutathione peroxidase [EC:1.11.1.9], and glutathione S-transferase [EC:2.5.1.18]) at 36 HAI. Although *TRAES3BF156400110CFD_g* and *TRAES3BF053500110CFD_g* (glutathione S-transferase [EC:2.5.1.18]) showed downregulation in B36 vs. A36, their FPKM was far lower than that of DEGs in L-ascorbate peroxidase [EC:1.11.1.11]. Therefore, L-ascorbate peroxidase [EC:1.11.1.11] might be critical in wheat seedling establishment in response to parental nitrogen fertilizer level.

**Figure 6:**
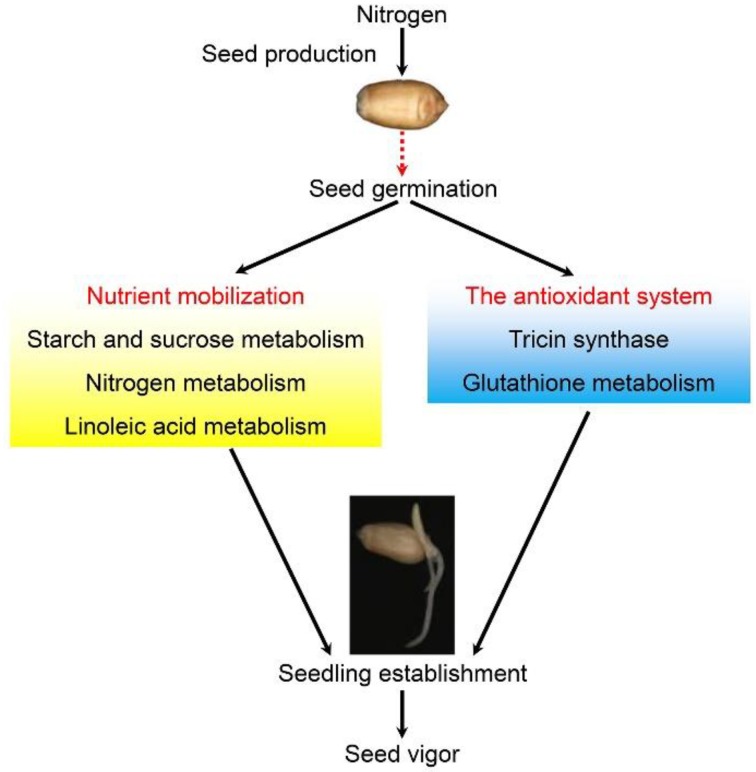
A possible network of nitrogen level during seed production affecting seedling establishment and seed vigor. Red dotted line indicates transition.

In the GO enrichment analysis, the number of GO terms in B36 vs. A36 was apparently more than that in EB36 vs. EA36. Moreover, the results of the KEGG enrichment analysis were similar to the GO enrichment analysis. These results indicated that changes of metabolism pathways in seeds also played important roles in seedling establishment under different parental nitrogen levels. The metabolism pathways in seeds are mainly involved in nutrient mobilization and the antioxidant system, such as starch and sugar metabolism, nitrogen metabolism, and glutathione metabolism. Therefore, nitrogen fertilizer level during seed production might affect the accumulation of multiple nutrients in seeds, thereby impacting seedling establishment and seed vigor. Taken together, we propose a possible network of nitrogen level during seed production affecting seedling establishment and seed vigor (Figure 6). Nitrogen fertilizer level significantly affects high-vigor seed production. Compared with nitrogen deficiency, seeds produced under a suitable nitrogen level show the upregulation of pathways involved in nutrient mobilization (starch and sugar, nitrogen and linoleic acid metabolism) and the antioxidant system (tricin synthase and glutathione metabolism), which promotes seedling establishment. Rapid seedling establishment displays high seed vigor.

In conclusion, nitrogen fertilizer affected not only grain yield but also seedling establishment and seed vigor in wheat. Pathways involved in nutrient mobilization and the antioxidant system played a pivotal role in seedling establishment and seed vigor in response to nitrogen level during seed production. Thus, this study provides new insights into seedling establishment in response to parental nitrogen level and could potentially be used to guide high-vigor seed production.

## 4. Materials and Methods

### 4.1. Materials

Wheat cultivar Tainong18 was grown in field trials in Dongwu Village (35°57′ N and 117°3′ E, Tai’an City, Shandong Province, China) during the period of wheat growth (October–June) in 2013–2014 and 2014–2015. This area has a semi-humid continental temperate monsoon climate [28]. The soil type is sandy loam, and the soil mineral N (NO^3–^ and NH^4+^) in the top 100 cm of soil before sowing is listed in Table S4.

### 4.2. Field Experiment

The trials were conducted with three replications using the randomized block design. Each field plot consisted of 12 lines. The length and width of each field plot was 40 and 3 m, respectively. Four nitrogen fertilizer levels, namely N0 (0 kg/ha), N168 (168 kg/ha), N240 (240 kg/ha, the usual nitrogen fertilizer level for winter wheat production in the North China Plain), and N300 (300 kg/ha), were used in this study. P (calcium superphosphate) and K (potassium chloride) were applied at rates of 120 kg/ha P_2_O_5_ and 75 kg/ha K_2_O, respectively. Total phosphate fertilizer, 40% nitrogen fertilizer, and 60% potash fertilizer were used as base fertilizer, and the residual 60% nitrogen fertilizer and 40% potash fertilizer were applied as topdressing at the jointing stage. We sowed seeds on 12 October 2013 and 12 October 2014, respectively. The plant density was 450 plants per m^2^. Seeds were harvested on 8 June 2014 and 10 June 2015, respectively.

### 4.3. Evaluation of Seed Vigor

Standard germination tests were performed according to the International Seed Testing Association with some modifications [36]. Fine silica sand with a diameter of 0.05–0.8 mm was used as a sprouting bed. Base sand consisted of 4-cm-height silica sand with 60% saturation moisture content per germination box (17 cm length, 11 cm width, and 7 cm height). One hundred seeds were sowed on the surface of the base sand, and then the seeds were covered with 2-cm-height silica sand with 60% saturation moisture content. All germination boxes were placed in a growth chamber at 20 ± 1 °C, 70% relative humidity, illumination conditions of 4000 lux, and a 24-h light photoperiod for 8 days. Each treatment consisted of three field plot replications and four technical replications per year. The lids of the germination boxes were removed at 3 days after imbibition (DAI), and then seedlings were sprayed with water of about 20 mL/day from germination 4 DAI. At 8 DAI, seedlings were washed to remove silica sand. Subsequently, the remaining seeds were removed from the seedlings. We recorded the number of seedlings per day, which were used to calculate the vigor index (VI) and germination index (GI). GI = ∑ (G_t_/D_t_), where G_t_ is the number of the germinated seeds on day t and D_t_ is the time corresponding to G_t_ in days. VI = GI × S, where S is plant fresh weight. VI was applied to evaluate seed vigor.

### 4.4. Seed Nitrogen Content

The semi-micro-Kjeldahl method was used to detect seed nitrogen content [19].

### 4.5. α-Amylase Activity

For α-amylase activity, 30 germinated seeds samples were ground with 15 mL deionized water and moderate silica sand. After extracting 20 min at room temperature, it was centrifuged at 3000× *g* for 10 min. Then, the supernatant was diluted to 100 mL with deionized water. The solution was used to determine α-amylase activity by the 3, 5–dinitrosalicylic acid method [37,38].

### 4.6. RNA Sequencing

Fifty germinated seeds and/or seedlings were pooled together as one biological sample for each treatment, which included three biological replicates. Samples were frozen in liquid nitrogen and then stored at −80 °C. Frozen samples were ground with liquid nitrogen in a mortar. Subsequently, total RNA was extracted by using the RNA extraction kit DP441 (Tiangen, Beijing, China). To avoid possible contamination and degradation, RNA was checked on 1% agarose gels. Moreover, a NanoPhotometer spectrophotometer (IMPLEN, Westlake Village, CA, USA) was used to examine RNA purity. RNA integrity was evaluated using the RNA Nano 6000 Assay Kit of the Bioanalyzer 2100 system (Agilent Technologies, Santa Clara, CA, USA). RNA concentration was detected by using Qubit RNA Assay Kit in Qubit 2.0 Flurometer (Life Technologies, Carlsbad, CA, USA).

RNA-seq library construction was performed by using NEBNext Ultra™ RNA Library Prep Kit for Illumina (NEB, Ipswich, MA, USA). Index codes were added to attribute sequences of each sample. An Illumina Hiseq platform was used to sequence the libraries and generate 150 bp paired-end reads. Raw reads of fastq format were preprocessed to remove low quality reads, reads containing adapter, and reads containing ploy-N. All the downstream analyses were based on high-quality clean reads. Clean reads were mapped to the wheat genome sequence (ftp://ftp.ensemblgenomes.org/pub/release-25/plants/fasta/triticum_aestivum/dna/) by using TopHat v2.0.12 [39]. HTSeq v0.6.1 was used to count the read numbers of each gene. Based on the length of the gene and read counts mapped to this gene, the FPKM (expected number of Fragments Per Kilobase of transcript sequence per Millions base pairs sequenced) of each gene was calculated. DESeq R package (1.18.0) was used to perform differential expression analysis of two groups [40,41]. Subsequently, the resulting *p*-values were adjusted by using Benjamini and Hochberg’s approach for controlling the false discovery rate and genes were assigned as differentially expressed with an adjusted of *p*-value < 0.05.

The GOseq R package was used to perform GO enrichment analysis of DEGs [42]. They were considered significantly enriched GO terms with a corrected *p*-value < 0.05. KOBAS software was used to test the statistical enrichment of DEGs in KEGG pathways [43].

### 4.7. qRT-PCR

Primer 6 software was used to design primers for qRT-PCR, and then primers were synthesized by Sangon Biotech (Shanghai, China). Gene specific primers are listed in Table S5. cDNAs were reverse transcribed from total RNA by using the PrimeScript RT reagent Kit (Takara, Dalian, China). All qRT-PCR analyses were performed on an ABI Stepone plus Real-Time PCR System (Applied Biosystems, Foster City, CA, USA) and each qRT-PCR experiment was repeated three times. An internal control, the wheat *Actin* gene, was used as to normalize the expression data [44]. The 2^−△△*C*t^ method was used to calculate the relative expression level of genes [45].

### 4.8. Statistical Analysis

SPSS 19.0 software (SPSS, Inc., Chicago, IL, USA) was used to perform one-way analysis of variance, Duncan’s multiple tests, and independent-samples T tests.

## Figures and Tables

**Table 1:** List of selected genes for glycerophosphodiester phosphodiesterase activity that were differentially expressed in B36 vs. A36.

Gene ID	Gene Annotation	log2FoldChange	*p*-Value
Traes_3DS_650F1C4CE	Protein WVD2-like 1	1.2020	2.43 × 10^−2^
Traes_2BL_56779CC56	Glycerophosphodiester phosphodiesterase GDPD2	0.9879	2.12 × 10^−2^
Traes_3AL_DABE7199F	Glycerophosphodiester phosphodiesterase GDPD1	0.9733	1.06 × 10^−13^
Traes_3DL_01383BD6A	Glycerophosphodiester phosphodiesterase GDPD1	0.9025	4.61 × 10^−5^
TRAES3BF012100010CFD_g	Glycerophosphodiester phosphodiesterase GDPD1	0.8868	3.36 × 10^−13^
Traes_2DL_4ADA8D564	Glycerophosphodiester phosphodiesterase GDPD2	0.6904	2.69 × 10^−2^
Traes_7AS_29FF84C97	Glycerophosphodiester phosphodiesterase GDPDL4	−0.5253	3.30 × 10^−3^

**Table 2:** List of selected genes for dioxygenase activity that were differentially expressed in EB36 vs. EA36.

Gene ID	Gene Annotation	log2FoldChange	*p*-Value
Traes_2DL_D4BCDAA76	Linoleate 9S-lipoxygenase 1	3.4937	1.65 × 10^−4^
Traes_2DL_CE85DC5C0	Linoleate 9S-lipoxygenase 1	3.1855	8.76 × 10^−3^
Traes_2DL_B5B62EE11	Probable linoleate 9S-lipoxygenase 5	2.7110	1.08 × 10^−14^
Traes_3DL_441FB3597	1-aminocyclopropane-1-carboxylate oxidase 3	2.4389	4.83 × 10^−3^
Traes_1AS_F64BAC19D	Protein SRG1	1.8862	2.33 × 10^−3^
Traes_2AL_BCC5296F4	Naringenin,2-oxoglutarate 3-dioxygenase	1.7767	4.41 × 10^−2^
Traes_4AL_D0DECE300	Feruloyl CoA ortho-hydroxylase 2	1.6432	2.18 × 10^−26^
Traes_1BS_CE840DC06	Protein SRG1	1.6176	3.87 × 10^−4^
Traes_4DS_A89A8FAD91	Gibberellin 20 oxidase 1	1.6130	9.08 × 10^−6^
Traes_5AL_76853D2CB	Putative linoleate 9S-lipoxygenase 3	1.4563	1.32 × 10^−2^
Traes_6DS_7CA5A8F12	Lipoxygenase 2.3, chloroplastic	1.4549	1.31 × 10^−6^
Traes_4BS_A772DDBD7	Gibberellin 20 oxidase 1	1.3838	7.84 × 10^−7^
Traes_2DS_6789FA5E7	1-aminocyclopropane-1-carboxylate oxidase homolog 1	1.3571	1.67 × 10^−2^
Traes_2DL_5E0E44CA3	Gibberellin 2-beta-dioxygenase 8	1.3534	2.73 × 10^−5^
TRAES3BF118400050CFD_g	Gibberellin 2-beta-dioxygenase 1	1.2993	1.20 × 10^−3^
Traes_2BL_1B1358201	Gibberellin 2-beta-dioxygenase 8	1.2264	1.55 × 10^−2^
Traes_5AL_C4A6AB34A	Linoleate 9S-lipoxygenase 1	1.2134	3.27 × 10^−2^
Traes_2BL_77148B8D8	Probable linoleate 9S-lipoxygenase 5	0.9053	1.49 × 10^−4^
Traes_5DS_E8892706A	Lipoxygenase 2.1, chloroplastic	0.8505	3.36 × 10^−2^
Traes_2BS_EE7040CA5	DIBOA-glucoside dioxygenase BX6	0.7073	9.60 × 10^−3^
Traes_2AL_5BAB26827	Seed linoleate 9S-lipoxygenase-3	0.6158	1.43 × 10^−3^
Traes_5BL_304FAFA26	Putative linoleate 9S-lipoxygenase 3	0.4723	8.39 × 10^−4^
Traes_2AS_768355513	DIBOA-glucoside dioxygenase BX6	0.3972	1.55 × 10^−2^
Traes_2AL_987F244D2	Potassium transporter 1	−0.5500	4.24 × 10^−3^

## Supplementary Materials

The following are available online at http://www.mdpi.com/1422-0067/19/11/3417/s1, Table S1: Summary of RNA-sequencing data, Table S2: Glutathione metabolism in KEGG enrichment, Table S3: Starch and sucrose metabolism in KEGG enrichment, Table S4: The soil mineral N (NO^3−^ and NH^4+^) (kg/ha) in the top 100 cm of soil profile before sowing at the period of wheat growth (October–June), Table S5: Primers used for qRT-PCR.
