# Supplementary material for: Effects of Nitrogen Level during Seed Production on Wheat Seed Vigor and Seedling Establishment at the Transcriptome Level
Source: Int J Mol Sci. 2018 Oct 31;19(11):3417. doi: 10.3390/ijms19113417 (PMC6274887; doi:10.3390/ijms19113417)
Supplement: Supplementary file 1 [file ijms-19-03417-s001.pdf]

Table S1. Summary of RNA-sequencing data

Sample	Raw Reads	Clean reads	Total mapped	Multiple mapped	Uniquely mapped
A36_1	41055620	39268722	29921951 (76.20%)	3937279 (10.03%)	25984672 (66.17%)
A36_2	43300282	41283562	31173850 (75.51%)	4092815 (9.91%)	27081035 (65.60%)
A36_3	47810934	45525014	34217489 (75.16%)	4458912 (9.79%)	29758577 (65.37%)
B36_1	52357806	49983548	37718470 (75.46%)	4976217 (9.96%)	32742253 (65.51%)
B36_2	49475938	47212956	35644906 (75.50%)	4620766 (9.79%)	31024140 (65.71%)
B36_3	50687470	48316388	36704968 (75.97%)	4810163 (9.96%)	31894805 (66.01%)
EA36_1	54740742	52296826	40166348 (76.80%)	5167060 (9.88%)	34999288 (66.92%)
EA36_2	58305732	55674718	42493785 (76.33%)	5487324 (9.86%)	37006461 (66.47%)
EA36_3	51060696	48793258	37273443 (76.39%)	4825924 (9.89%)	32447519 (66.50%)
EB36_1	48699118	46511436	35635831 (76.62%)	4604049 (9.90%)	31031782 (66.72%)
EB36_2	43988474	42024340	32007231 (76.16%)	4097597 (9.75%)	27909634 (66.41%)
EB36_3	43518306	41424378	31745682 (76.64%)	4192764 (10.12%)	27552918 (66.51%)

Table S2. Glutathione metabolism in KEGG enrichment

EC	Gene ID	Log2Foldchange	FPKM-N240	FPKM-N0
B36 vs A36				
Glutathione S-transferase [EC:2.5.1.18]	Traes_4DL_469179461	0.8990	12.97	7.21
	TRAES3BF156400110CFD_g	-0.5987	70.65	110.97
	TRAES3BF053500110CFD_g	-1.2455	2.78	6.83
L-ascorbate peroxidase [EC:1.11.1.11]	Traes_4DL_8CE055F15	0.8765	498.33	281.40
	Traes_4BL_FBE8A057A	0.5248	492.57	354.99
	Traes_4AS_9EEABCE1C	0.7054	390.61	248.33
	Traes_4BL_19FA6DCAD	0.4494	316.53	240.33
EB36 vs EA36				
Glutathione synthase [EC:6.3.2.3]	Traes_7DL_CCBA1A1C9	0.7602	6.17	3.73
Glutathione peroxidase [EC:1.11.1.9]	Traes_6DL_F16253948	0.6236	36.65	24.37
	Traes_6BL_8360C77EF	0.8595	9.52	5.37
Glutathione S-transferase [EC:2.5.1.18]	Traes_4DL_469179461	1.2496	10.44	4.50
	Traes_1AS_CBD6D1EA5	-1.4347	1.28	3.54
L-ascorbate peroxidase [EC:1.11.1.11]	Traes_4DL_8CE055F15	0.6995	487.19	307.66
	Traes_4BL_FBE8A057A	0.4202	450.68	345.48
	Traes_4AS_9EEABCE1C	0.4877	379.95	277.79
	Traes_4BL_19FA6DCAD	0.4201	315.58	241.87

Table S3. Starch and sucrose metabolism in KEGG enrichment

Ec	Gene ID	Log2Foldchange	FPKM-N240	FPKM-N0
B36 vs A36				
Starch phosphorylase [EC:2.4.1.1]	Traes_3DL_FFCCD5827	0.4230	48.12	37.20
Alpha-amylase [EC:3.2.1.1]	Traes_6DL_5BE701A64	0.5238	2766.56	1995.15
	Traes_6DL_E31AB6EED	0.5511	2247.18	1590.26
	Traes_6BL_8115FDC31	0.6470	1849.65	1224.79
	Traes_7AL_3C8D25CCD	-0.5676	162.80	250.22
	Traes_7BL_0687BD4F8	-0.9060	60.73	118.04
Beta-glucosidase [EC:3.2.1.21]	Traes_4AL_0BE08D907	0.3506	150.18	122.07
	Traes_5AL_133FEF770	-0.9259	6.49	12.78
Beta-fructofuranosidase [EC:3.2.1.26]	Traes_2BL_3EDC425A7	0.4994	11.91	8.73
EB36 vs EA36				
Beta-glucosidase [EC:3.2.1.21]	Traes_5BL_AA69820C5	0.4417	209.21	157.80
	Traes_5DL_C3F949BDC	0.4835	93.88	68.79
	Traes_5AL_BADD20945	0.6241	57.18	38.01
	Traes_4DS_24F6CC544	0.5767	35.20	24.19
	Traes_2BS_2EDCB210E	0.5343	28.85	20.41
Beta-fructofuranosidase [EC:3.2.1.26]	Traes_4AL_16456D1B6	0.6160	52.42	35.06
	Traes_2BL_3EDC425A7	0.6413	20.57	13.52
	Traes_2AL_6DEEE3C3E	0.9482	14.15	7.51
	Traes_4AL_6E7713B2F	0.5749	6.16	4.23
	Traes_6DS_204661A07	-0.6570	5.87	9.49
Beta-D-xylosidase 4 [EC:3.2.1.37]	Traes_2DL_7799F1F75	1.8695	14.32	4.02
Pectinesterase [EC:3.1.1.11]	Traes_1AL_1C66B5A24	-0.8401	2.85	5.23

Table S4. The soil mineral N (NO^{3-} and NH^{4+}) (kg/ha) in the top 100 cm of soil profile before sowing at the period of wheat growth (October – June).

Treatment	2013 – 2014	2014 – 2015
N0	65.00 ± 3.83 d	83.30 ± 5.16 d
N168	146.56 ± 16.33 c	121.20 ± 2.64 c
N240	207.10 ± 12.36 b	165.20 ± 11.39 b
N300	262.57 ± 31.42 a	275.06 ± 18.73 a

Different letters indicated significant differences among means under different treatments (p value < 0.05 by one-way ANOVA analysis).

Table S5. Primers used for qRT-PCR.

Primer	Sequence (5'to 3')
Actin-F	CGAAGCGACATACAATTCCATC
Actin-R	GAACCTCCACTGAGAACAACAT
Traes_6BL_8115FDC31-F	CGCACACGGCAATGACTATG
Traes_6BL_8115FDC31-R	GCTCGGACCAATATGTATCACG
Traes_4DL_469179461-F	CGCTTGGATTGCCAGGAACTA
Traes_4DL_469179461-R	CCTCGTTGTTATGCTCCAGTGA
Traes_3AL_DABE7199F-F	GGTGTGATGTCCTTCGGTGAG
Traes_3AL_DABE7199F-R	ACGGTCCATTGTGACGGTGA
Traes_7BL_0687BD4F8-F	CCTCCAGCGATCCACCATTC
Traes_7BL_0687BD4F8-R	TCGTGTGAGCAATTTGTAGAGC
TRAES3BF113400140CFD_g-F	TCCTGCTGCTGCTCTTCCTT
TRAES3BF113400140CFD_g-R	CGGCGGGTAGTAGACGATGA
Traes_2BL_232323148-F	CGCCTCAGCCGTGTGATAGT
Traes_2BL_232323148-R	GCTAGACGCTAGAAGCACCAAC
Traes_3DL_441FB3597-F	CGTCCCTGGAACTGGAAACAAA
Traes_3DL_441FB3597-R	GACATACTACACGAGTCGGCAT
Traes_5BL_60FC12DA6-F	GCCGCCTCTCAATACGACGAT
Traes_5BL_60FC12DA6-R	CGAAGACGCCATGACACCCT
Traes_7DS_26341EE29-F	ATCGGCGTCCTCTCTGATGTC
Traes_7DS_26341EE29-R	CGTTCACCTTGGCGTTCTCC
Traes_6DL_5B3132521-F	GACCTCTCCTCCAACAATCTCT
Traes_6DL_5B3132521-R	AACACCTCCACCTGGTATCTG
Traes_1BL_A0980046F-F	CCTGGTGGATTCAACCGATGT
Traes_1BL_A0980046F-R	GACTCGTCGTTAGCATTCCTCT
Traes_5DL_B6AAC8E52-F	CACTGGACTTCTTGCTGGATTC
Traes_5DL_B6AAC8E52-R	CGATGTCTAGGTAGGTGAGGTT
